# Can evidence-based health policy from high-income countries be applied to lower-income countries: considering barriers and facilitators to an organ donor registry in Mumbai, India

**DOI:** 10.1186/s12961-016-0075-6

**Published:** 2016-01-13

**Authors:** Diana K. Vania, Glen E. Randall

**Affiliations:** 1 Global Health, Faculty of Health Sciences, McMaster University, 1280 Main Street West, DSB 509, Hamilton, ON L8S 4M4 Canada; 2 Health Policy & Management, DeGroote School of Business, McMaster University, 1280 Main Street West, DSB 227, Hamilton, ON L8S 4M4 Canada; 3 Centre for Health Economics and Policy Analysis, McMaster University, 1280 Main Street West, CRL Building 282, Hamilton, ON L8S 4K1 Canada

**Keywords:** India, Organ donation, Organ donor registry, Policy analysis

## Abstract

**Background:**

Organ transplantation has become an effective means to extend lives; however, a major obstacle is the lack of availability of cadaveric organs. India has one of the lowest cadaver organ donation rates in the world. If India could increase the donor rate, the demand for many organs could be met. Evidence from high-income countries suggests that an organ donor registry can be a valuable tool for increasing donor rates. The purpose of this study is to determine whether the implementation of an organ donor registry is a feasible and appropriate policy option to enhance cadaver organ donation rates in a lower-income country.

**Methods:**

This qualitative policy analysis employs semi-structured interviews with physicians, transplant coordinators, and representatives of organ donation advocacy groups in Mumbai. Interviews were designed to better understand current organ donation procedures and explore key informants’ perceptions about Indian government health priorities and the likelihood of an organ donor registry in Mumbai. The 3-i framework (ideas, interests, and institutions) is used to examine how government decisions surrounding organ donation policies are shaped.

**Results:**

Findings indicate that organ donation in India is a complex issue due to low public awareness, misperceptions of religious doctrines, the need for family consent, and a nation-wide focus on disease control. Key informants cite social, political, and infrastructural barriers to the implementation of an organ donor registry, including widely held myths about organ donation, competing health priorities, and limited hospital infrastructure.

**Conclusions:**

At present, both the central government and Maharashtra state government struggle to balance international pressures to improve overall population health with the desire to also enhance individual health. Implementing an organ donor registry in Mumbai is not a feasible or appropriate policy option in India’s current political and social environment, as the barriers, identified through the 3-i framework lens, may be too difficult to overcome. Despite the evidence supporting the use of donor registries as a means to enhance organ donation rates, it is clear that context is critical and that it is not always practical to apply evidence-based policy solutions from high-income countries to lower-income settings.

**Electronic supplementary material:**

The online version of this article (doi:10.1186/s12961-016-0075-6) contains supplementary material, which is available to authorized users.

## Introduction

Organ transplantation has become an effective means to save and extend lives; however, a major obstacle is typically the lack of availability of cadaveric organs. Many factors contribute to the unavailability of organs internationally, including lack of knowledge about organ donation, difficulty obtaining familial consent, and insufficient hospital infrastructure [1,2]. Research in high-income countries has shown that one of the ways in which the cadaver organ donation rate can be increased is the implementation of an organ donor registry [3,4]. A donor registry allows residents of a particular region to declare their willingness to donate their organs after death. Maintaining a donor registry creates public awareness about post-mortem organ donation and allows healthcare providers to demonstrate to families that their relative wished to become an organ donor [2,4]. Implementation of a donor registry is often associated with an increase in publicity about organ donation, leading to a more informed population, thus positively affecting organ donation rates [1,5]. However, based on our review of the literature, very little research has been conducted on organ donation in lower-income countries, so it remains unclear the extent to which a policy solution used in high-income countries may be practical [6].

India has one of the lowest cadaver organ donor rates in the world at just 0.08 donors per million population per year [7]. In many other countries, cadaver donor rates typically range from 10 to 25 donors per million population [8]. Current estimates indicate that more than 275,000 kidneys, livers, and hearts are required in India, but less than 2% of people in need receive an organ [9]. The number of people in India requiring transplants is expected to increase due to the rising burden of chronic illnesses, such as diabetes, which can cause issues with multiple vital organs, further exacerbating the need for organ transplants [10]. If India could increase the cadaver donation rate to just one donor per million population per year, this would meet the current demand for all livers, hearts, lungs, and some kidneys for the entire country [11].

The purpose of this study is to determine whether the implementation of an organ donor registry is a feasible and appropriate policy option to enhance cadaver organ donation rates in a lower-income country. In addition, this paper describes current organ donation procedures, explores key informants’ perceptions about Indian government health priorities, and examines how Indian government decisions surrounding organ donation policies are shaped by ideas, interests, and institutions within the health policy context, while balancing the need to improve both population and individual health.

## Background

### Organ donation policies in India

In 1994, India’s Ministry of Health and Family Welfare implemented the Transplantation of Human Organs Act [12]. The purpose of the act was to regulate the “*removal, storage and transplantation of human organs for therapeutic purposes*” and to prevent commercialization of organs in India ([12], p. 1). Since 1994, the Act has been revised three times. The most important change comes from the Transplantation of Human Organs (Amendment) Bill, 2009, which aims to increase the number of cadaveric organs harvested from brain-dead patients by requiring a doctor in an intensive care unit (ICU) to inform families about organ donation and obtain consent for donation [13]. Although the government has implemented and refined India’s transplant policies, other major policies or programs, such as the implementation of a donor registry, have not been put in place as a means to increase the supply of cadaver organs.

## Methods

A case study of organ donation in Mumbai was conducted through one-on-one semi-structured interviews with physicians, transplant coordinators, and representatives of organ donation advocacy groups in Mumbai in 2013. This study received ethics approval from the McMaster University Hamilton Health Sciences/Faculty of Health Sciences Research Ethics Board in January 2013.

This study focused only on Mumbai; as the largest city in India, it is more likely that Mumbai has sufficient infrastructure to effectively develop and implement a donor registry. The city’s resources and diverse and cosmopolitan nature make it difficult to generalize findings to other parts of Maharashtra or India. Smaller cities may not have the adequate resources and infrastructure to perform organ transplants. This study was conducted in the district of Mumbai City (as defined by the 2011 Indian Census) [14]. Hospitals in this district that perform transplants are registered with Mumbai’s Zonal Transplant Coordination Center (ZTCC), an organization that promotes cadaver organ donation and oversees the distribution of cadaver organs.

Individual interviews were conducted with key informants from private and government hospitals and non-governmental organizations within Mumbai. The development of the interview guide was informed by earlier work, which included a review of the literature and review of government documents related to organ donation [6]. Each interview lasted about 40 minutes. Interviews were designed to better understand current organ donation policies and procedures and examine key informants’ perceptions about whether the implementation of an organ donor registry is a feasible and appropriate policy option to enhance cadaver organ donation rates in Mumbai and Indian government health priorities (see Additional file 1 for interview guide). A purposive sampling strategy was employed in order to select participants who are knowledgeable about health policy and who are involved in organ donation in Mumbai, so as to gain maximum representation of views from the identified groups (physicians, transplant coordinators, and organ donation advocacy groups). Individuals without a high level of policy-related expertise would find it difficult to answer the specific questions being posed about the implementation of an organ donor registry. A possible future study might consider the views of patients and non-experts. In order to ensure the highest level of expertise of potential participants, only physicians and transplant coordinators of Mumbai hospitals registered with and authorized by the ZTCC to perform organ transplants were contacted. In addition, the sampling strategy was supplemented with snowball sampling, in which study participants were asked to provide names of other potential participants. A Letter of Information was provided to participants to explain the project and interview process and a Consent Form was signed prior to the interviews.

Interviews were transcribed for key informants who agreed to have their interview recorded. Detailed notes were taken during the other interviews. Analysis of notes and interview transcripts occurred concurrently with data collection. With this approach, the researcher continually refined and modified interview questions based on previous participant responses and explored themes as they emerged from interviews [15]. Once all interviews had been conducted, the complete transcripts were read by the primary researcher for a preliminary impression of responses. Constant comparison was employed throughout data analysis, in which a transcript is compared within itself, against other interviews with similar participants, and against interviews from different groups. This method of qualitative analysis allows the researcher to determine if study participants’ responses are similar or divergent and to identify similarities and differences between groups of respondents [16].

Data was divided into sections based on a predetermined framework derived from the study aims and topics raised by respondents. This deductive approach allowed the primary researcher to use a pre-set structure to analyse transcripts and interview notes [16]. Interview data was then indexed by marking the transcripts and categorizing responses into themes and sub-themes that arose. The general principles from the consolidated criteria for reporting qualitative research (COREQ) framework was used to guide the reporting of qualitative research [17].

## Results

Interviews were conducted with 15 key informants, including eight physicians who are involved in organ transplantation: four nephrologists, one urologist, one gastroenterologist, and two cardiologists; five transplant coordinators (three are educated as medical social workers, two are trained as physicians); and two representatives of organizations involved in facilitating organ donation in Mumbai. Ten participants were male and five were female; the physicians were heavily represented by males, while the majority of transplant coordinators were represented by females.

Interview respondents discussed factors that influence organ donor rates in Mumbai, outlined transplant data and procedures in Mumbai, identified actors who have an interest in organ donation, and examined institutional structures that may hinder the advancement of the transplant program in Mumbai. Framed within the ideas, interests, and institutions framework, major topics and themes raised by key informants were identified and explored. These included myths and misperceptions about religious doctrines, public awareness and education, issues around the transplant waiting list, low level of government interest, limited hospital infrastructure and capacity, issues around family consent, and the role of foreign institutions in domestic policy development. Each of these will be discussed below.

### Ideas – knowledge, beliefs, and values

A key finding of the research was that knowledge, beliefs, and values influence organ donation rates in Mumbai. Important factors identified included myths and misperceptions of one’s religious teachings, the lack of public awareness and education about organ donation, and the transplant waiting list process.

#### Myths and misperceptions about religious doctrines

Myths are an impediment to garnering support for organ donation. Lack of awareness about organ donation criteria, procedures, and familial preferences often result in families not giving permission to retrieve their relative’s organs. It was reported that some families may worry that “*there is scare of disfigurement, or that you should have the whole body at time of cremation*” [Respondent 2 (R2)]. Other families may fear that the harvested organs will be sold instead of being transplanted into a wait-listed recipient. In donation after brain death, the observed practice in Mumbai, the “*heart is still beating so* [families] *feel that the patient is still alive, or maybe* [there is] *chance of survival*” [R2]. Transplant coordinators and physicians believed these perceptions should be addressed in public awareness campaigns to properly inform people about organ donation.

Several key informants asserted that, even if a donor registry was implemented in Mumbai, garnering public support for organ donation and increasing the cadaver donor rate would still be difficult due to objections on religious grounds. Respondents argued that, despite the fact that all major religions in Mumbai consider organ donation an acceptable practice, many individuals may not recognize this and may still object to organ donation on the basis of religion. One key informant noted that “*all religions support organ donation. But* [people] *may not be aware that their religion* [supports it]” [R2]. Another participant stated that “*if the religious leaders, the spiritual leaders consent that organ donation is important, definitely* [people] *will take the initiative*” [R9].

#### Awareness and education

Some respondents saw merit in introducing a donor registry, as it would enhance awareness about organ donation, but a few felt it would not necessarily directly lead to an increase in donor rates. The vast majority of key informants asserted that more direct public awareness campaigns about organ donation are needed to make people more receptive to the idea of organ donation, which could help increase the cadaver donor rate in Mumbai. Some key informants reported that current efforts to spread awareness (lectures, newspaper articles, donor felicitation ceremonies) are not adequate, and that large-scale media campaigns are required to garner support for organ donation across the city. Some respondents argued that public awareness is the only way to increase support for organ donation. One key informant contended that, while a registry may have cosmetic value, it would not be productive or cost-effective at this time; rather, money should be spent on outreach and “[sensitizing] *normal people to the thought of organ donation*” [R1].

As well as educating the general public about organ donation, some participants indicated that educating healthcare providers is also an important step to increasing organ donation. One transplant coordinator felt that physicians who are not involved in transplants may not support organ donation because they are not aware of the potential benefits. This respondent believed this lack of physician education could hinder the expansion of the transplant program. A physician respondent noted that targeting “*ICU doctors, ICU nurses, social workers in the hospital who are in charge of transplant coordination and making them aware*” [R1] is important because they are the ones directly involved in convincing families to consent to donation.

#### Transplant waiting lists in Mumbai

**Fig. 1:**
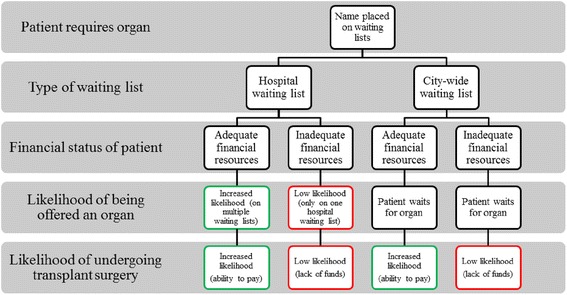
**Likelihood of receiving an organ in Mumbai.** Patients with adequate financial resources have an increased likelihood of receiving an organ. When a patient in Mumbai requires an organ, their name is placed on both the hospital waiting list and the city-wide waiting list. Although financial status of the patient is not considered when determining an organ recipient, the likelihood of being offered an organ is greatly improved for those who are wealthier and able to place their name on multiple hospital waiting lists. The likelihood of undergoing transplant surgery is also increased for those who are able to pay for the surgery.

In 2013, at the time data was collected for this study, 2,523 people in Mumbai were waiting for a kidney and 136 were waiting for a liver. However, one respondent noted that between January and June 2013, Mumbai had only 11 cadaver donors, which translated to 20 kidneys and 11 livers. Kidneys are the most sought-after organ because India has “*the largest diabetic population in the world* [and] *kidney failure is a huge problem*” [R8]. Patients who require a kidney are placed on two waiting lists: the hospital waiting list and a city-wide waiting list with the ZTCC. Because of the fragmented transplantation system, patients are able to register themselves on multiple hospital kidney waiting lists to improve their chances of receiving a kidney. One respondent explained, “*So what patients do is go and put their names in many hospitals to take advantage of that. So, suppose my patient feels that his number is a bit low in my* [hospital] *list, he’ll go to a newer hospital where there are less patients, put his name there also*” [R1]. While key informants stated that, although the order of the waiting list is always adhered to and no patient can pay for higher priority on any individual waiting list, patients who can afford to place their name on multiple hospital waiting lists (by visiting different nephrologists in various hospitals) have a higher chance of receiving an organ. Figure 1 illustrates that patients with adequate financial resources have an increased likelihood of receiving an organ.

When a patient is declared brain-dead in a registered hospital and the family has consented to organ removal for donation, the ZTCC is contacted to oversee organ allocation. As per standard practice in Mumbai, the liver and one kidney are reserved for the retrieving hospital (where the patient died) if there is a compatible recipient on that hospital’s waiting list. If there is no compatible recipient at the retrieving hospital, the organs are allocated to the city-wide waiting list. The second kidney and all other organs, including the heart, pancreas, and lungs, are directly allocated to the next compatible recipients on the city-wide waiting list. If there is no compatible recipient in Mumbai, the organ will likely go to waste, as there is no inter-state sharing program in place to distribute organs to other states.

**Fig. 2:**
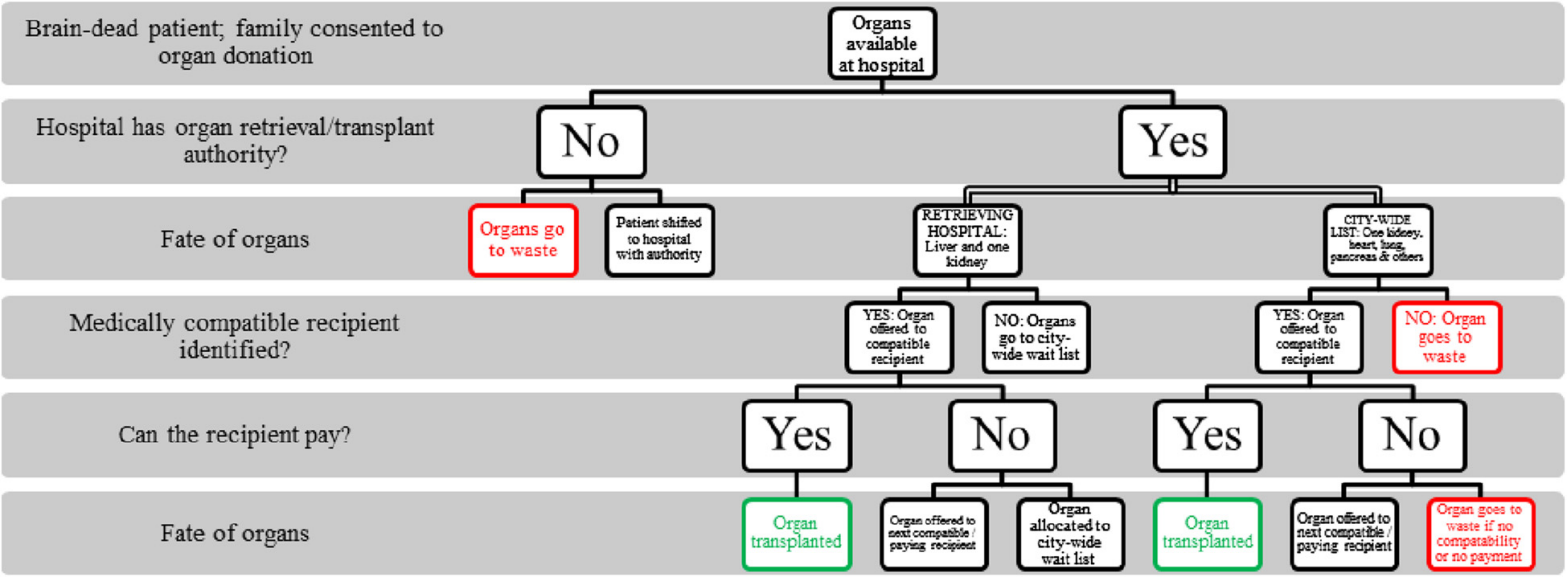
**Organ allocation process in Mumbai.** As per standard practice in Mumbai, the liver and one kidney are reserved for the retrieving hospital (where the patient died) if there is a compatible recipient. If there is no compatible recipient at the retrieving hospital, the organ is given to the city-wide waiting list. All other viable organs, including one kidney, the heart, pancreas, and lungs are allocated to recipients on the Mumbai-wide waiting list. If there are no compatible recipients in Mumbai, the organ will likely go to waste, as there is no inter-city sharing program in India.

According to key informants, although placement on a waiting list does not take into account a patient’s financial or socioeconomic status, actually receiving an organ is based on ability to pay rather than on distribution equity principles. Therefore, although the waiting list is technically blind to financial status, for practical purposes, the system does by-pass the poor if they are not in an immediate position to pay for the operation. Only medically relevant criteria are considered when ranking patients for the waiting list; although “*financial criteria is not considered when we’re listing the* [waiting list] *scoring*” [R10], potential recipients are asked if they are financially prepared to undergo the transplant surgery. If a patient is unable to pay for the transplant, he or she will be passed over and the organ will be allocated to the next compatible recipient who is able to pay. The flow chart in Fig. 2 shows the organ allocation process in Mumbai. This diagram illustrates the complex procedure for allocating a cadaveric organ in Mumbai, as well as showing that patients who are able to pay for surgery are more likely to undergo an organ transplant than patients who are unable to do so.

### Interests – actors and organizations

Many actors, including the government, physicians, and transplant coordinators, contribute to shaping organ donation in Mumbai. It is important to consider the roles that executives (government) and healthcare professionals play in order to determine how those with more power and higher status often have more influence on policy decision-making.

#### Government interest

Several respondents suggested that the introduction of the Transplantation of Human Organs Act, 1994, resulted in more standardized and transparent organ transplantation procedures in India. Apart from this legislation, both the state and national governments’ perceived lack of priority on organ donation was identified by respondents as a barrier to improving the organ donor rate, and the need for a donor registry will not likely gain their attention. Most key informants stated that organ donation has “*not become the priority because* [India] *as a country, we are facing problems. We are having people dying of cholera, tuberculosis…transplant sort of becomes* [lower priority]. *So actually basic health issues are the priority to the government*” [R2].

#### Physicians and transplant coordinators

Strict criteria are set out for organ transplantation staff and institutions. Physicians who declare brain death must be registered with the government to be involved in transplants. One interviewee stated that it is important for physicians to be registered before declaring brain death because “*the worry here is that if you ask any physician to declare brain death, there’ll be no control over who is declared, and the last thing that an early transplant program needs is a scandal*” [R3].

Transplant coordinators play a critical role in attaining family consent for organ donation. They are responsible for liaising with ICU patients and their families, educating families on organ donation, and informing the ZTCC when there is a potential brain-dead patient. The transplant coordinator builds a rapport with the families and attempts to determine the patient’s views on organ donation: “*a good hospital transplant coordinator will make a round of intensive care units and see who are potential donors and will remain in touch with the intensive care team*” [R12].

One participant contrasted transplant coordinators in private hospitals to those working in government hospitals. The participant believed that transplant coordinators in private hospitals are committed to their role and are sincerely dedicated to counselling families, encouraging organ donation, and identifying potential donors to help other patients. This participant felt that, in contrast, government hospital transplant coordinators regard their role completely differently. The interviewee stated that medical social workers in government hospitals are not sensitized to the benefit of organ donation, such as saving the lives of up to eight people, and as government servants, “*they’re just doing it for the sake of doing it; it’s a government job*” [R10].

### Institutions – procedures and rules

#### Hospital infrastructure and capacity

A major barrier to increasing the number of retrieved cadaver organs is the transplant capacity and infrastructure of hospitals in Mumbai; healthcare staff and resources are often limited. Most respondents indicate that private hospitals typically perform more transplants because they have sufficient facilities, whereas government hospitals do not: “*there’s only one government hospital at the moment which does liver transplant…while about six to seven private hospitals* [offer] *it*” [R3]. Government hospitals have trauma centres and see more traffic accident victims, and therefore “*they are likely to get more potential donors*” [R13], but most government hospitals in Mumbai do not have adequate authority, technical capacity, and human resources to maintain brain-dead patients and retrieve and transplant organs.

One physician informant believed that, without improvements to hospital infrastructure and transplant capacity in Mumbai, organ donation cannot thrive in the city, indicating that because only select hospitals in large cities in India currently have the capacity and authorization to perform transplants, up to “*90 or 95% of the potential donors* [are wasted] *simply because the facilities to…identify them as donors, get the infrastructure in place, even to actually do the transplant harvest, that facility exists in only few centres*” [R7].

#### Family consent

In India, medical decisions are typically made jointly by families rather than by individuals. This cultural norm extends to decisions about organ donation. Even if the patient had expressed a desire to become an organ donor by signing a donor card, the family still makes the final decision at the time of brain death, so their knowledge of and consent to organ donation is critical. Key informants indicate that most rejections come from families who “*are not knowing about the concept of brain death*” [R2] and from families who wonder “*whether something can be done for the* [brain-dead] *patient*” [R3]. Respondents stated that as awareness about organ donation increases, families are more likely to consent to organ donation, and that some families are beginning to approach doctors asking if they can donate their relative’s organs.

## Discussion

This research assesses the feasibility of developing and implementing an organ donor registry in Mumbai, India. Key informants indicated that the current organ donation procedures in Mumbai are complicated and that public awareness and knowledge of organ donation is low in Mumbai, both amongst the public and the government. Input from key informants illustrated that both organ donation policy and broader health policy development are shaped by ideas, interests, and institutions within India. An assessment of ideas, interests, and institutions can be helpful when analysing policy issues and potential implementation of a new policy. These elements are discussed below in an examination of current organ donation policies in Mumbai, possible reasons why the Indian and Maharashtrian governments have not taken long-term action to increase the cadaver donor rate, and subsequently, whether the implementation of an organ donor registry is a feasible and appropriate policy option.

### Ideas – knowledge, beliefs, and values

In its broadest sense, ideas encompass the elements of knowledge, evidence, beliefs, and values. Analysing the role of ideas in policy development is important because it provides insight into how each of the elements promotes or inhibits policy change. Although knowledge and research evidence on their own are not always sufficient to lead to new or reformed policies, they can help modify beliefs over time. In contrast, changing core values is much more difficult, as values tend to be deeply ingrained in individuals (and across societies) and resist change even in the face of strong evidence.

#### Knowledge and evidence

The organ donation system in India consists of a complex maze of programs and procedures, and there is a lack of research about organ donation in India. Most organ donation research in India centres on medical tourism and organ commercialization. Only recently have researchers started conducting research that focuses on addressing the needs of those on a transplant waiting list. Disseminating information to the masses will require involvement from various interest groups, including physicians and transplant coordinators, as well as the media and religious leaders.

Mumbai’s literacy rate and diversity of languages spoken must be taken into account when considering education programs and a donor registry. The city’s literacy rate is 89%, which means that approximately 1.3 million people cannot read or write beyond a minimal level [14]. Surveys have shown that the illiterate have the highest rate of being opposed to organ donation, which poses several challenges for spreading awareness about organ donation [18]. First, information dissemination must clearly outline the risks, benefits, and options of organ donation. Second, different mediums must be used to reach and enlighten the general population. Third, in order to facilitate full understanding of organ donation and its process, a registry should include translation into multiple languages. In addition to Hindi and English (the nation’s official languages) and Marathi (the state language), translation is required in many other languages, as the city’s cosmopolitan nature and large influx of migrants result in as many as 16 major languages being spoken in Mumbai [19].

Policy change and increased funding toward organ donation will likely only occur with a combination of comprehensive research evidence, a strong advocate for organ donation, and when the government views organ donation as a sufficiently important issue. That being said, education campaigns about organ donation are vital to begin shifting the perceptions of the public. Previous studies have indicated that public awareness campaigns that refute organ donation myths and perceived consequences are effective in increasing intent to become an organ donor after death [20,21]. Even if the supporting policies and infrastructure are not yet in place, changing the inaccurate beliefs about organ donation can improve the likelihood that people will consent to post-mortem organ donation.

#### Beliefs

Misperceptions about religious doctrines are a major impediment to garnering support for organ donation and increasing the cadaver donor rate. Study findings are consistent with the literature, which states that there is a distinct discord between religious teachings and peoples’ perceptions of what their religion allows [22]. In Mumbai, none of the major religions prohibit donating one’s organs after death, and even the religions that initially state a preference against organ donation, change their stance when taken holistically against the backdrop of helping others [18,23]. With the appropriate knowledge and evidence and support from religious leaders, beliefs may be influenced and the city may see higher donation rates even in the absence of a donor registry.

#### Values

The values held by policy actors and established institutions influence policy choices. In the Indian context, international organizations’ and the central government’s emphasis on improving population health through targeting communicable diseases affects the advancement of the transplant program. Larger population health issues often enter the government agenda and organ donation cannot gain priority.

The Indian government struggles with balancing international directives to improve overall population health with the need to improve individual health. In comparison to population-wide health initiatives addressing communicable diseases, which could positively affect millions of people, the beneficiaries of organ transplants are relatively few. Increasing the cadaver donor rate aims to improve individual health rather than overall population health. Realistically, however, the government should be concerned about improving both individual health and population health; these two concepts influence each other and are not mutually exclusive. There needs to be a balance between appreciating the need for large-scale population-wide programs and smaller-scale initiatives that will aid in the betterment of quality of life for those who can afford the treatment. It may well be that the predominant role of the private sector in the delivery of individual health services has lessened the Indian government’s sense of responsibility for delivering services deemed to be the purview of the private sector. Ultimately, the Indian government will need to find a way to balance between maximizing equality (population health) and optimizing individual well-being (through services such as a transplant program), especially as evidence is emerging that there will be increased need for transplants in the near future.

### Interests – actors and organizations

Policy actors are guided by ingrained values and the institutions surrounding them. Those who have a stake in a certain issue or policy will usually work towards ensuring that the development or effects of a policy will benefit themselves in some way. Common interests can mobilize groups to attempt to influence policymakers, but it is typically the actors or organizations with more power and money that have greater influence on shaping policies. Those who actually use the services and have more need for them may have very little power in policy decision-making. Groups who have a vested interest in organ donation policies in Mumbai include the government, healthcare providers, and patients. Even within these groups, there are divided interests based on the structures and resources that surround them.

#### Government

Unsteady and wavering support for organ donation from the central government indicates to state governments and organizations that organ donation may not be a priority for the country. In January 2012, the Ministry of Health and Family Welfare announced plans to enhance the National Organ Transplant Programme with the aim to increase the number of cadaver donors and improve organ retrieval capacity in hospitals [24]. However, like many issues in a country so fraught with disease, the issue faded away and the policy window closed as more pressing concerns entered the government’s policy agenda [25]. As a result, no tangible action was taken on the part of the government to take advantage of this fleeting focusing event and, only a few months later, the budget for the National Organ Transplant Programme was cut by over 90% and the Ministry renounced their support for an enhanced transplant program [26].

In a country besieged by epidemics, pandemics, and a rise in non-communicable diseases, the National Organ Transplant Programme will mostly likely not be a priority for the current Indian government or their successors. The brief placement of organ donation on the policy agenda suggests that the government is aware of the need for improvements to the transplant program, but that other health issues are more pressing at this time. While evidence suggests that the transplant program is advancing through updates in legislation and the plan to introduce organ donor stickers on ID cards in Maharashtra, key informant responses indicate that organ donation is not a priority for the government due to other health issues facing the country. This implies that organ donation is still on the government agenda, but is not a top priority.

#### Physicians and transplant coordinators

Physicians in private hospitals (where most organ transplants in Mumbai occur) may have higher interests in improving their own transplant program than in improving the city-wide transplant program. Some organ transplants do occur in municipal and government hospitals, but due to inadequate resources, medical staff often make the difficult choice to focus on treating and saving patients instead of maintaining brain-dead patients for transplant purposes. Thus, the organ transplant priorities of private hospitals are higher than transplant priorities of public hospitals, simply because private hospitals have more resources to focus on organ donation. At best, the development of a donor registry may help streamline the organ retrieval and transplant process, but it is unlikely that a registry will be considered a priority by physicians broadly. Physicians who do support the development of a registry will most likely be transplant surgeons and specialists working in private hospitals, since their patients and hospital would benefit most from an increase in available organs.

Transplant coordinators, on the other hand, may have a stronger interest in the development of an organ donor registry. The main role of the transplant coordinator is to encourage families to donate their relative’s organs, and this process might be facilitated with the knowledge that the patient already joined the donor registry. Similar to physicians, transplant coordinators in private hospitals may show stronger support for the development of a registry, as patients in private hospitals would benefit more than patients in public hospitals.

#### Patients

Despite India being the world’s largest democracy, which could be associated with the opportunity for the public to effect change, it is unlikely that the general population will focus on the need to increase the organ donor rate. Rather, only people directly affected by the lack of cadaver organs for transplant (wait-listed patients, recipients, and families) would be expected to advocate to increase awareness about organ donation and to improve the donor rate. Although transplant wait-list patients would benefit from an increased cadaver donor rate, it is unlikely that this small sub-population will mobilize to greatly affect donation policies in Mumbai. Transplant patients are a diffuse group spread across a city of over 12 million people afflicted by other health concerns; it is difficult for small patient groups to organize and effectively demonstrate any degree of power in influencing policies in Mumbai. Therefore, it is unlikely that a donor registry will gain the support of the general public right now, as the population who receives the benefit is very small.

### Institutions – procedures and rules

Institutions refer to the procedures for developing policies. These procedures can be considered the set of rules that guide processes and behaviours of policy decision-makers. Policy legacies, in which governments are “*predisposed towards policies with which they already have some favourable experience*”, are strong influencers of subsequent policy decisions ([27], p. 11). In India, the policy legacy of targeting communicable diseases affects the ability of other health issues, such as organ donation, to gain attention on the policy agenda. It is important to take into account India’s past policy decisions and structural constraints when analysing policymaking and current organ donation policies.

#### Policy legacy – communicable diseases

Since the 1950s and 1960s, India’s health sector has been engrossed in eradicating communicable diseases [28]. The long-standing focus on infectious diseases, without very much attention being paid to burgeoning health issues (such as the various chronic diseases now affecting India’s population), has led to the present-day health system still being very focused on communicable disease initiatives. The emphasis on providing short-term solutions to infectious diseases rather than addressing the underlying causes of diseases is further exacerbated by the increasing role of private actors in public health policy who financially support large-scale public health initiatives to eliminate infectious diseases. Under this stance, it is unlikely that an organ donor registry will come to fruition, as it does not advance the established efforts of communicable disease eradication.

#### Foreign actors

External aid agencies and foundations provide significant health financing, for which India must comply with guidelines and work towards the external funders’ priorities, almost singularly focus on infectious diseases and decreasing the global burden of disease. Near the end of the 20^th^ century, there was a shift in health policymaking in which public-private partnerships gained momentum, taking over the role of United Nations agencies. International organizations are not only funding health programs, but are also setting health policies and may have a more influential role in setting national health policies than do the countries’ own politicians [29]. It is in India’s best interests to follow the directives of these funding agencies in order to continue receiving financing, rather than focus on improving cadaver organ donation rates, which has very little to no international or national government funding support. As long as large international agencies are channelling vast sums of money into Indian health programs, the interests of these influential actors will take precedence over organ donation in India.

### Study limitations

This study has three main limitations that should be acknowledged. First, this study focused only on Mumbai; results cannot be generalized to the state of Maharashtra or to the whole of India. As the largest city in India, it is more likely that Mumbai is closer to having the infrastructure required to successfully execute a transplant program. However, because of Mumbai’s size and urban resources, study findings may not be relevant to extrapolate to smaller cities, which may not have the human resources, hospital and technical capacity, or financial means to perform organ transplants and develop a donor registry. Second, because only participants from Mumbai were interviewed, their perspectives cannot be generalized to healthcare providers in other towns and cities. Physicians and transplant coordinators who work in Mumbai, India’s most cosmopolitan city, may be more liberal-minded and open to changes in organ donation procedures. A large-scale study with participants from a mix of both large and smaller cities needs to be conducted. Finally, key informants were selected based on purposive sampling, which may lead to volunteer bias. This can affect the reliability of the study as results may not be consistently reproduced, as well as the validity of the study since participants who agreed to participate in the study may be more open and willing to talk about organ donation than the general population.

## Conclusions

Like many countries around the world, the demand for organs in India far outstrips the supply. This analysis has revealed that implementing an organ donor registry in Mumbai is not a feasible or appropriate policy option, as the barriers, identified through the ideas, interests, and institutions lens, may be too difficult to overcome in India’s current political and social environment.

The probability of an issue reaching and remaining at the top of the Indian government policy agenda is affected by the complex interplay among numerous actors and organizations, more widespread health issues competing for scarce resources, and the broader political environment within which health policies are considered. In the absence of a focusing event or a high profile policy entrepreneur who is able to champion the cause and push the issue of availability of organs for transplant onto the policy agenda, the government may have little incentive to take up the cause in more than a superficial way. Taken together, this assessment strongly supports the contention that there will be no political appetite for any government-sponsored organ donation policy initiatives despite the demonstrated need.

A registry would require infrastructure and resources that are currently not available, especially when there is not widespread support for organ donation. Even if the general public was aware of the registry and willing to donate their organs, due to illiteracy and lack of access to technology, many in Mumbai would not be able to register. At best, implementing a registry without addressing other organ donation issues could make the donation process more efficient by determining who was a willing potential donor prior to the family being approached by a transplant coordinator; however, given the current social and political environment, it is unlikely that a registry alone would lead to an increase in donor rates in Mumbai without addressing concurrent issues.

Policy change is most likely to occur when ideas, interests, and institutions align, providing a political and social environment conducive to change. Given the current situation in Mumbai of little substantive research evidence, lack of strong political advocates for organ donation, and powerful policy legacies, it is unlikely that organ donation will make it to the top of the government policy agenda and that an organ donor registry will be implemented. Moreover, despite the evidence supporting the use of donor registries as a means to enhance organ donation rates, it is clear that context is critical and that it is not always practical to apply evidence-based policy solutions from high-income countries to lower-income settings.

## Additional file

Additional file 1:
**Interview guide.** (PDF 104 kb)
