# Supplementary material for: Can evidence-based health policy from high-income countries be applied to lower-income countries: considering barriers and facilitators to an organ donor registry in Mumbai, India
Source: Health Res Policy Syst. 2016 Jan 13;14:3. doi: 10.1186/s12961-016-0075-6 (PMC4712496; doi:10.1186/s12961-016-0075-6)
Supplement: Additional file 1: — Interview guide. (PDF 104 kb) [file 12961_2016_75_MOESM1_ESM.pdf]

Appendix A: Interview Guide

1. Currently, what is the procedure to receive an organ in Mumbai if someone is in need?
    a. What works and what does not work in this process? What changes are needed?
2. What is the current number of organs needed versus organs available?
3. What are the factors that influence a patient's position on the organ transplant wait list?
    a. Is the rank in wait list adhered to?
4. What is the procedure to ask families about donating their relative's organs after death?
    a. What steps are taken to convince families to consent to donation?
    b. Are families usually agreeable to consenting to donation?
5. How is information shared between hospitals about organs available for transplantation?
6. Is there priority for organs to go to the wealthy or those who are able to pay?
7. Do patients in private hospitals have higher priority for receiving an organ?
8. What efforts have been made to increase awareness about organ donation, both among the public and within the government?
    a. Have governments, health care providers, and the public been receptive to these messages?
9. What is the government doing to minimize illegal organ donation?
10. How does medical tourism affect the Indian economy?
11. Who has jurisdiction over the health system? (National, state, municipal)
    a. How does this affect organ transplants?
12. Is organ donation a priority for the government?
13. Is organ donation a priority for your organization?
14. What might some barriers be to developing an organ donor registry? (*i.e., religious, political, cultural*)
15. Do you think that religious beliefs would be a barrier to garnering support for organ donation?
16. What resources would be needed to implement and make use of an organ donor registry in your organization? (*i.e., time, money, personnel, technology*)
    a. Does your organization have the resources that are needed?
17. What kind of donor registry would work best in Mumbai (opt-in or opt-out)?
